# Polarization-insensitive stimulated Brillouin scattering filter with birefringence compensation

**DOI:** 10.1371/journal.pone.0357986

**Published:** 2026-09-16

**Authors:** Jing Li, Xiangguan Tan, Jiaqing Liu, Yan Xu

**Affiliations:** 1 College of Electronics and Information Engineering, Shandong University of Science and technology, Qingdao, Shandong, P.R China; 2 Ceyear Electronics Technology Instruments Co., Ltd, Qingdao, Shandong, P.R China; Rutgers University Newark, UNITED STATES OF AMERICA

## Abstract

Stimulated Brillouin scattering (SBS) has been extensively studied and repurposed for diverse practical applications, such as optical measurement and sensing, and microwave photonics filter. SBS based optical filters face inherent limitations due to polarization-dependent gain fluctuations caused by fiber birefringence and environmental perturbations. To address these challenges, we establish a comprehensive theoretical model of polarization evolution in the fiber and implement depolarization via the Faraday effect. Building on this insight, we propose and experimentally validate a polarization-independent SBS filter architecture enabled by dual orthogonally polarized pumps generated through a Faraday rotator mirror (FRM)-integrated Mach-Zehnder interferometer (MZI)**.** This design achieves passive compensation for polarization fluctuations without requiring active or complex polarization state control. Experimental validation confirms that this design suppresses Brillouin gain variations to less than ±3% degree of polarization, enabling wavelength-independent operation across arbitrary input polarizations. In contrast, existing approaches based on complex polarization control schemes typically exhibit a degree of polarization exceeding ±5% and are susceptible to variations in both operating wavelength and filter parameters. Furthermore, we demonstrate the filter’s versatility in optical spectrum analysis (OSA) using a two-stage SBS configuration, achieving a remarkable dynamic range of 80 dB, ± 0.3 pm wavelength precision, and improved power accuracy from ±2.4 dB to within ±0.2 dB, ensuring rapid and precise spectral acquisition. These advancements resolve long-standing limitations in SBS-based systems, enabling robust and polarization-independent operation for optical communications and sensing, integrated photonic devices and circuits, optical neural networks, microwave photonics, passive and active photonic devices.

## Introduction

Stimulated Brillouin Scattering (SBS) requires the lowest activation power among all nonlinear effects in optical fibers. Although SBS-induced backward scattering degrades system performance in optical communication and sensor systems, necessitating its suppression in such applications. On the other hand, SBS has also been extensively studied and repurposed for diverse practical applications. For instance, by leveraging SBS in kilometer-long fibers or centimeter-scale integrated photonic waveguides, researchers have developed SBS-based optoelectronic devices for distributed sensing, optical communication, optical storage, optical metrology, microwave photonic, optical neural networks, Brillouin lasers, and nonreciprocal transmission, among others [1–4]. Specific examples include optical filters, optical signal processors, optical signal amplification, pulse compression, waveform synthesis, and slow-fast light generation for photonic control and generation [5–7]. Beyond optical systems, SBS has enabled RF functionalities such as microwave photonics filter, phase shifters, delay lines, frequency measurement systems and RF beamforming [8–10], while also enhancing high-capacity optical communications [11,12].

However, a critical limitation of SBS lies in its polarization dependence. Horiguchi first explored the relationship between SOP alignment and SBS gain; later, detailed analyses of optimal polarization conditions were provided. Their work confirmed that SBS amplification in fibers is intrinsically polarization-dependent [13,14]. This polarization-dependent response is a critical factor in practical single-mode fibers, as intrinsic birefringence inevitably alters the SOP during light propagation. Consequently, the overall SBS gain (or loss) depends on both the fiber’s birefringent properties and the input SOPs of the pump and signal. Building on this understanding, several methods based on polarization adjustment or pump depolarization via depolarizers have been proposed [1–4]. However, these methods suffer from various limitations. For instance, they may fail when the signal’s SOP changes or may be limited to a small wavelength coverage [13–15].

In this study, we aim to eliminate the polarization dependence of the SBS effect itself. We first present a practical model to describe polarization fluctuation effects caused by linear and nonlinear birefringence in optical fiber. Building on this foundation, we propose, analyze and experimentally validate a polarization- and wavelength-independent SBS-based tunable filter. Central to this design is the elimination of complex polarization state control by integrating a Faraday rotator mirror (FRM) into a power-splitting Mach-Zehnder interferometer (MZI) structure. This configuration generates dual orthogonal pumps, effectively suppressing polarization fluctuations in the SBS process induced by fiber birefringence. Notably, when the MZI parameters are optimized, the degree of polarization (DOP) of the pump source is reduced to less than ±3%.

To demonstrate the filter’s advanced capabilities, we apply it in ultra-high-resolution optical spectrum analysis. The results reveal that the filter can accurately perform ultra-high-resolution measurements on signals with arbitrary polarization states. By reducing the polarization-dependent gain from over 20 dB to less than 1 dB, the spectral measurement stability shows significant improvement, with power uncertainty within ±0.2 dB, and an 80 dB dynamic range via adaptive baseline correction is achieved. Finally, a sweep speed of 20 nm/s for the tunable laser source (TLS) enables a high spectral resolution of 0.08 pm, with wavelength precision maintained within ±0.3 pm.

In summary, this work overcomes the shortcomings of conventional approaches by realizing a novel SBS-based narrowband tunable filter that is insensitive to both wavelength and polarization. This advancement delivers significant improvements in critical system performance.

Compared with scalar SBS-based filters, the polarization- and wavelength-insensitive filter offers higher selectivity and an elevated depletion threshold. Beyond its use in ultrahigh-resolution spectral analyzers, it is also highly attractive for applications such as selecting sub-bands in next-generation ultrahigh-capacity optical communication and optical networks, microwave-photonic filtering of broadband RF signals, and serving as a key component in photonic integrated circuit, among others.

## Concept and operation principle

### Polarization fluctuation model in fiber and depolarization

In isotropic materials, the optical Kerr effect induces self-birefringence, resulting in a progressive rotation of the polarization ellipse during light propagation, a phenomenon known as polarization ellipse self-rotation [16]. However, optical fibers introduce additional complexity due to inherent localized birefringence and external perturbations, such as temperature gradients and mechanical pressure. These factors collectively make linear birefringence the dominant component of intrinsic fiber birefringence [17].

To rigorously model this phenomenon, we start with coupled nonlinear Schrödinger equations to describe light propagation within an optical fiber [16]. Under the assumptions of linear birefringence, low-loss conditions, and negligible polarization mode coupling effects, the evolution of the polarization vector is given by [18–20]:


$$\begin{array}{c} \partial_{z}\psi_{z}=-i(\omega B\sigma_{\theta }+\omega \alpha {\langle \sigma_{\text{3}}\rangle }_{\psi_{z}}\sigma_{\text{3}})\psi_{z} \end{array}$$ (1)

Here, $\psi_{z}=\text{\textbackslash{}hspace\{0.33em}\}(E_{\text{1}}\left(z\right),E_{\text{2}}\left(z\right){)}^{t}$ represents the Jones vector of the transverse electric fields at position z along the fiber. In the θ direction, the first term models linear birefringence, where σ_θ_ = σ_1_cos(θ)+σ_2_sin(θ) incorporates the 2 × 2 Pauli matrices with σ_1,2,3_. Here, ω is the optical frequency, B is the frequency-independent birefringence coefficient, and ωB is defined by phase birefringence. The second term accounts for nonlinear birefringence, with $\alpha =\frac{n_{\text{2}}P}{3cA_{eff}}$ where n_2_ is the nonlinear refractive index, A_eff_ is the effective mode area of the fiber, P is the total input power, c is the light speed, and ${\langle \sigma_{\text{3}}\rangle }_{\psi_{z}}=\frac{|E_{\text{1}}(z){|}^{\text{1}}-|E_{\text{2}}\left(z\right){|}^{\text{2}}}{|E_{\text{1}}\left(z\right){|}^{\text{1}}+|E_{\text{2}}\left(z\right){|}^{\text{2}}}$.

On the Poincaré sphere, the linear birefringence term drives a rotation of the Stokes vector around the equatorial axis σ_θ_, whereas the nonlinear term induces a vertical-axis rotation. Eq (1) shows that the angular velocity and orientation of this rotation depend on the instantaneous polarization state via ${\langle \sigma_{\text{3}}\rangle }_{\psi_{z}}$. Notably, in conventional fibers, linear birefringence dominates (B»α) even at high optical powers, while the vertical-axis rotational velocity remains negligible. For example, a fiber with a 20 m beat length exhibits B ≈ 0.25 ps/km versus α ≈ 0.00035 ps/km at 100 mW (λ = 1550nm, n_2_ = 3.2 × 10^−20^, A_eff_ = 60µm^2^) [21]. Consequently, the nonlinear term acts as a secondary perturbation to the dominant linear effect, a critical insight into SBS processes, where pump powers rarely exceed tens of milliwatts [1].

Reformulation Eq (1) by employing the identity $\psi_{z}={\langle \sigma \rangle }_{\psi_{z}}\sigma \psi_{z}$ clarifies this hierarchy, the phenomenon more apparent, reinforcing its theoretical basis.


$$\begin{array}{c} \partial_{z}\psi_{z}=-i\omega B\sigma_{\theta }\psi_{z}-i\omega \alpha \frac{\text{1}}{\text{2}}\left({\langle \sigma_{\text{3}}\rangle }_{\psi_{z}}\sigma_{\text{3}}+\left(1-{\langle \sigma_{\theta +\pi /2}\rangle }_{\psi_{z}}\sigma_{\theta +\pi /2}-{\langle \sigma_{\theta }\rangle }_{\psi_{z}}\sigma_{\theta }\right)\right)\psi_{z} \end{array}$$ (2)

Here, the term proportional to ψ merely influences the global phase and can therefore be neglected. Furthermore, the two terms ${\langle \sigma_{\text{3}}\rangle }_{\psi_{z}}\sigma_{\text{3}}$ and ${\langle \sigma_{\theta +\pi /2}\rangle }_{\psi_{z}}\sigma_{\theta +\pi /2}$ cancel each other out to first order, leaving only a residual second-order precession component in the instantaneous rotation axis. Consequently, this simplification yields an approximate evolution equation for the polarization vector [19,20]:


$$\begin{array}{c} \partial_{z}\psi_{z}\approx -i\omega B_{eff}\sigma_{\theta }\psi_{z} \end{array}$$ (3)

with the effective birefringence:


$$\begin{array}{c} B_{eff}=B-\frac{\alpha }{\text{2}}{\langle \sigma_{\theta }\rangle }_{\psi_{z}} \end{array}$$ (4)

The polarization vector is determined by both the intensity and polarization state of the light. Eq (3) conserves |ψ_z_|^2^, consistent with the lossless fiber assumptions in SBS. For a linearly polarized input, this result aligns with Stolen’s theoretical framework [22]. The explicit solution:


$$\begin{array}{c} \psi_{z}=exp(-i\omega B_{eff}\sigma_{\theta }z)\psi_{\text{0}} \end{array}$$ (5)

This reveals that polarization fluctuations arise from rotations around σ_θ_, which is proportional to B_eff_. ψ_0_ denotes the Jones column vector representing the input light, and the rotation angle β is defined as follows:


$$\begin{array}{c} \beta =\omega \left(B-\frac{\alpha }{\text{2}}m_{\theta }\left(0\right)\right)z \end{array}$$ (6)

Here, m_θ_(0) represents the component of the incident polarization vector aligned with the birefringence axis σ_θ_, while z denotes the longitudinal position relative to the fiber’s input end.

Eq (5) illustrates that even minimal variations in B dominate over nonlinear and intensity-related effects, as B»α under typical power conditions [23].

Consequently, reformulating the solution of Eq (3) to Stokes formalism, the polarization state at position z becomes:


$$\begin{array}{c} \overset{⌢}{S}\left(z\right)=\overset{\wedge }{R_{\theta }}\left(\beta \left(L\right)\right)\overset{⌢}{S}\left(0\right) \end{array}$$ (7)

Here, $\overset{⌢}{S}\left(0\right)$ represents the Stokes vector of the incident light, $\mathrm{\backslash stackrel}\wedge R_{\theta }$ is the rotation operator. Consequently, the SOP of the incident light exhibits fluctuations along its propagation within fiber.

**Fig 1:**
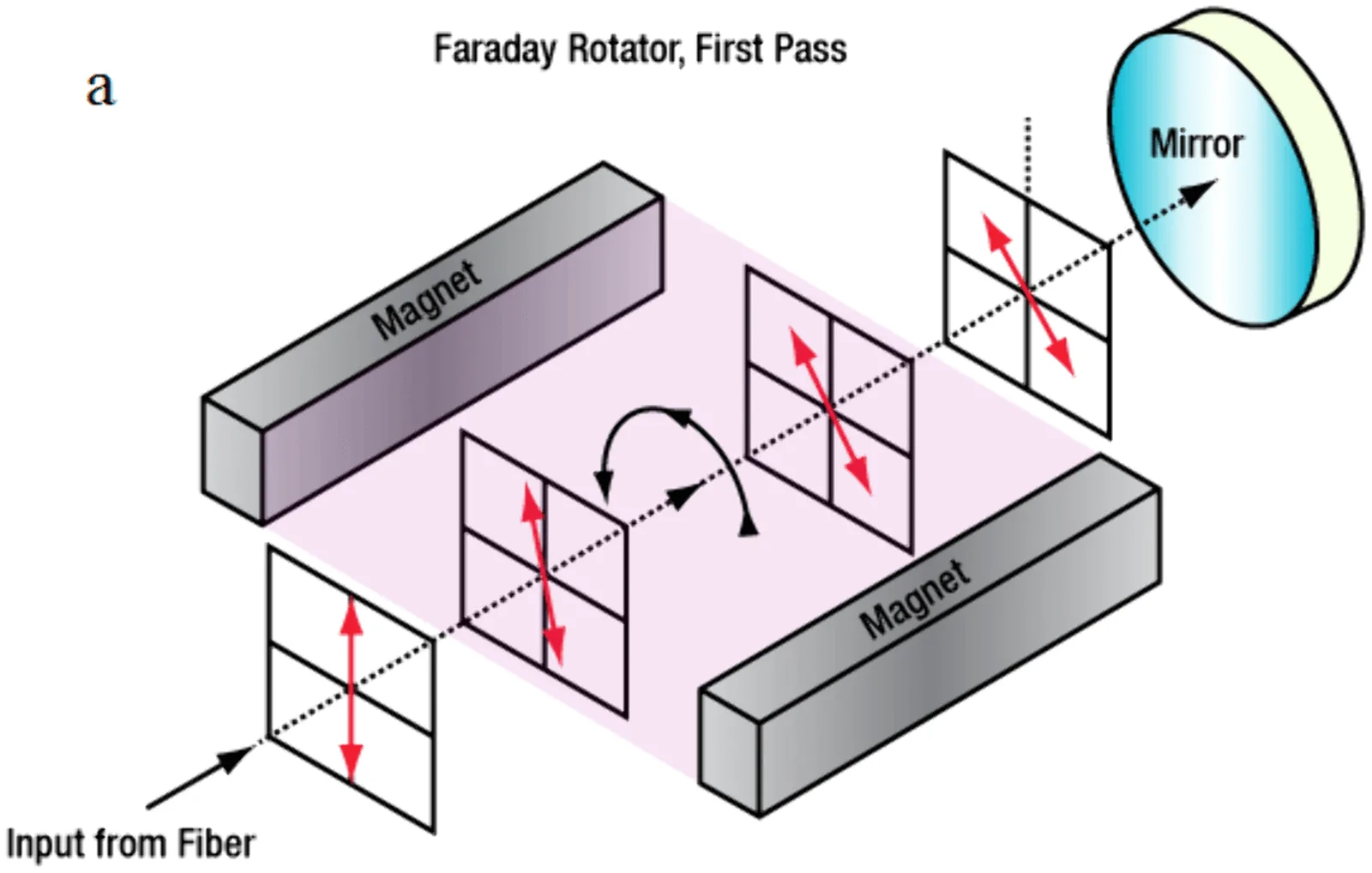
Polarization State of the input light of the FRM.

**Fig 2:**
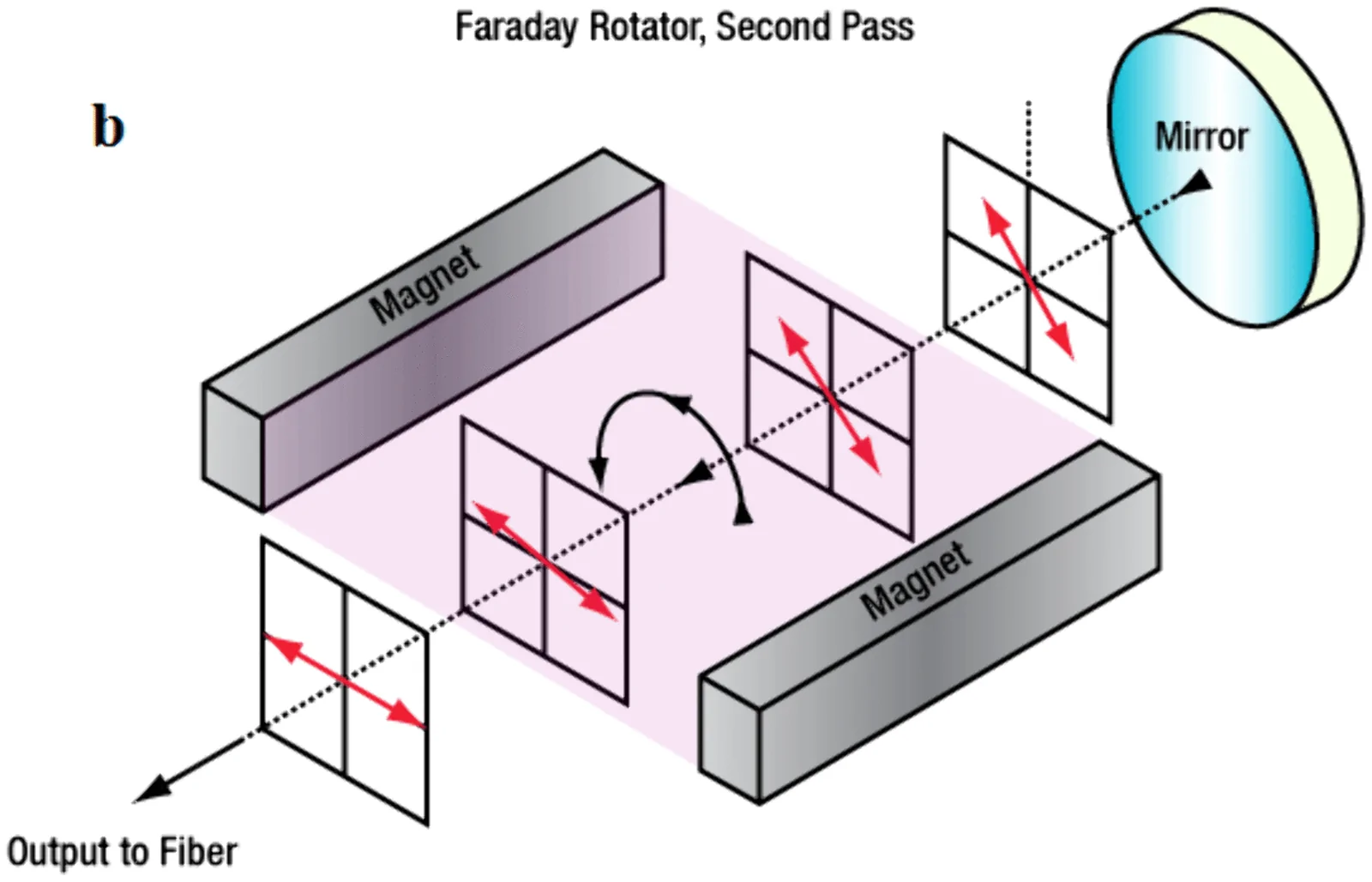
Polarization state of the output light of the FRM.


This framework confirms that polarization states fluctuate along the fiber due to birefringence, a critical challenge for SBS amplification, as unstable polarization alignment perturbs Brillouin frequency shifts and reduces gain efficiency [24]. To mitigate these instabilities, we implement a dual-pump architecture using orthogonally polarized pumps generated via a FRM. As shown in Fig 1 and Fig 2, the FRM imposes a 90^°^ polarization rotation upon reflection, ensuring orthogonality between forward and retro-reflected light components. In this scheme, the pump light is evenly divided by a 1 × 2 coupler: one segment light propagates through a segment of optical fiber to maintain polarization state stability as described by Eq (7), while the other propagates through a circulator toward the FRM and is later recombined. For the portion of light described by Eq (7) that interacts with the FRM, the Stokes vector undergoes a transformation:

$$\begin{array}{c} {\overset{⌢}{S}}^{F}\left(z\right)=-\frac{\text{1}}{\text{2}}\overset{⌢}{S}\left(z\right) \end{array}$$ (8)

The superscript F denotes the reversed polarization state by the FRM reflection.

The light is present, as demonstrated by Eqs. (7) and (8), combines, the copropagating dual orthogonal pumps in the SBS process induce mutual cancellation of intrinsic birefringence-driven polarization fluctuations. This cancellation leaves only residual nonlinear birefringence terms, which are sufficiently small to be treated as perturbative noise [25].

To mathematically describe the polarization states of the dual orthogonal pumps, we define them using unit Stokes vectors ${\overset{⌢}{S}}^{∥}$ and ${\overset{⌢}{S}}^{⟂}$, which serve as orthogonal basis vectors within this case. By leveraging these vectors, we can accurately analyze the interaction of the dual orthogonal pumps in the SBS process, providing a solid foundation for understanding and controlling the polarization-related phenomena in our experimental setup.

The SBS process with dual orthogonal pumps is mathematically formulated as follows: an arbitrarily polarized incident light can be decomposed into the orthogonal basis vectors ${\overset{⌢}{S}}^{∥}$ and ${\overset{⌢}{S}}^{⟂}$:


$$\begin{array}{c} \psi_{z}(\omega_{sig},z=0)=\psi_{\text{0}}\left(\omega_{sig}\right)(a{\overset{\mathrm{\backslash lower}0.5em\mathrm{\backslash smash}⌢}{\}}S}^{∥}+b{\overset{\mathrm{\backslash lower}0.5em\mathrm{\backslash smash}⌢}{\}}S}^{⟂}) \end{array}$$ (9)

$\psi_{\text{0}}\left(\omega_{sig}\right)$ constitute a spectrally varying, complex-valued scalar quantity, as $|a{|}^{\text{2}}+|b{|}^{\text{2}}=1$. Following Brillouin amplification, the output Stokes vector becomes [26]:


$$\begin{array}{c} \begin{array}{c} \psi_{z}(\omega_{sig},z=L)=\psi_{\text{0}}\left(\omega_{sig}\right)\left(aG_{max}\left(\omega_{sig}){\overset{\mathrm{\backslash lower}0.5em\mathrm{\backslash smash}⌢}{\}}S}^{∥}+bG_{max}(\omega_{sig}\right){\overset{\mathrm{\backslash lower}0.5em\mathrm{\backslash smash}⌢}{\}}S}^{⟂}\right)\\ =\psi_{\text{0}}\left(\omega_{sig}\right)G_{max}\left(\omega_{sig}\right)\psi_{z}(\omega_{sig},z=0) \end{array} \end{array}$$ (10)

Here, G_max_(ω_sig_) denotes the frequency-dependent max SBS gain coefficient (with the unit of m^-1^). It is derived from the convolution of the intrinsic SBS line shape and the pump’s power spectral density (PSD) [1]. Notably, the SBS gain spectrum achieves and maintains its maximum amplification under co-polarized pump-signal configurations, while orthogonal alignments suppress interaction, thereby enhancing the overall stability and reliability of the SBS-related operations.

To eliminate polarization-dependent gain instability, we implement a dual-pump architecture with orthogonally polarized pumps. In this scheme, the dual pump components propagate through the fiber with similar polarization fluctuations, preserving their mutual orthogonality. This inherent symmetry enables a self-stabilizing compensation mechanism, where fluctuations in one pump component are counterbalanced by those in the orthogonal counterpart, effectively canceling polarization-induced distortions. The mutual cancellation of polarization artifacts suppresses gain variations across arbitrary signal polarizations, thereby stabilizing amplification and eliminating polarization-dependent instabilities in the SBS process. This approach opens up new possibilities for more stable and efficient optical signal processing.

By replacing a single pump with an orthogonally polarized dual-pump configuration, polarization fluctuations are mutually canceled. As the two pump components propagate within the fiber, their birefringence-induced fluctuations evolve similarly while maintaining orthogonality, creating an intrinsic compensation mechanism. This approach ensures stable amplification of arbitrarily polarized incident light, free from polarization-dependent instabilities, and is expected to enhance critical performance metrics in Brillouin filtering applications, including spectral resolution and rejection ratios.

Conventional polarization adjustment-based methods, which involve numerous polarization controllers or rely on the polarization pulling effect of SBS amplification, may fail when the light’s SOP changes, a situation that frequently occurs in practice. Conventional approaches that depolarize the light using depolarizers are also limited, as depolarizers are polarization-sensitive and operate within narrow spectral bands, typically covering only a small wavelength range. In comparison, this paper demonstrates a polarization- and wavelength-independent SBS-based filter that achieves excellent performance by design, without requiring complex control mechanisms.

### Polarization-insensitive optical filtering and spectral analysis

To describe the principle of our proposed polarization-insensitive SBS-based optical filtering method, we begin by deriving the analytical expression for the amplified signal under steady-state conditions, using the well-known SBS three-wave coupled equations [27].

In this theoretical model, two counter-propagating optical beams, the pump and the signal, generate thermally excited acoustic phonons when specific polarization and frequency conditions are satisfied. The interaction between these beams initiates Brillouin scattering, resulting in frequency-shifted Stokes and anti-Stokes components. The backscattered light exhibits a Doppler frequency shift. Under steady-state conditions, the SBS amplified signal can be described by two partial differential equations [13, 21].


$$\begin{array}{c} \frac{dP_{p}\left(z\right)}{dz}=-\alpha P_{p}\left(z\right)-\frac{g_{B}\left(\omega \right)}{A_{eff}}P_{s}\left(z\right)P_{p}\left(z\right)-P_{esp}\left(z\right) \end{array}$$ (11)

$$\begin{array}{c} \frac{dP_{s}\left(z\right)}{dz}=\alpha P_{s}\left(z\right)-\frac{g_{B}\left(\omega \right)}{A_{eff}}P_{s}\left(z\right)P_{p}\left(z\right)-P_{esp}\left(z\right) \end{array}$$ (12)

Here, P_p_(z) and P_s_(z) represent the pump and signal intensities along the fiber, respectively, while P_esp_ accounts for the contribution of spontaneous Brillouin emission from thermally excited phonons. g_B_(ω) is the frequency-dependent Brillouin gain coefficient, A_eff_ is the fiber’s effective mode area, and α denotes the fiber attenuation coefficient.

**Fig 3:**
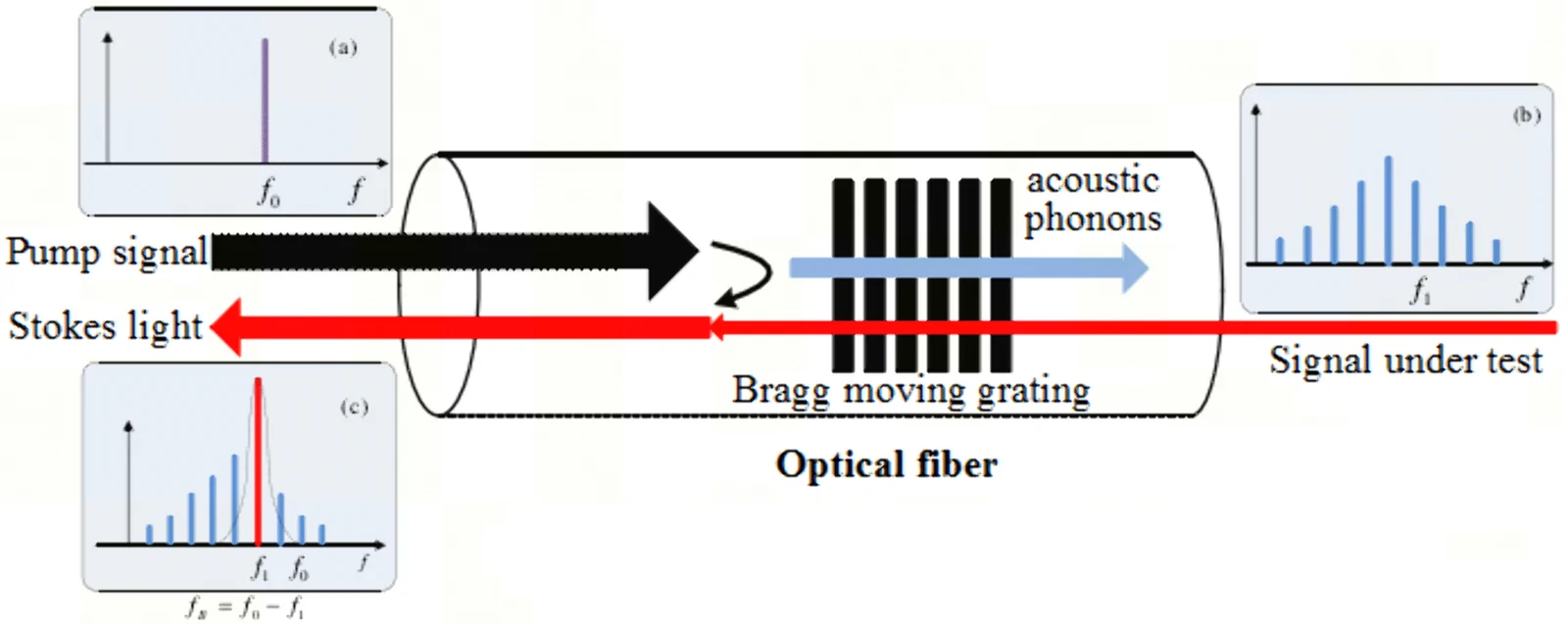
Principle of SBS-based spectrum analysis.


For spectral analysis applications, as shown in Fig 3, we consider a tunable continuous-wave (CW) pump operating at wavelength λ_p_, launched into a single-mode fiber of length L at z = 0. Concurrently, a wideband signal under test (SUT), P_s_, is injected at z = L. The SUT’s spectral components near the Brillouin-shifted wavelength λ_B_ = λ_p_ + Δλ_B_ undergo amplification within the Brillouin gain bandwidth. By sweeping the pump wavelength, specific spectral regions of the SUT can be selectively amplified, enabling reconstruction of its entire spectrum. From a spectrum analysis perspective, the SUT at z = L can be divided into in-band and out-of-band spectral regions:

$$\begin{array}{c} P_{s}\left(L,\lambda \right)=P_{s}\left(L,\lambda_{B}\right)+P_{s}(L,\lambda \neq \lambda_{B}) \end{array}$$ (13)

Assuming negligible fiber propagation losses, the backscattered output signal I_s_(z, λ) captured via an optical circulator at z = 0, comprises in-band and out-of-band spectral terms as well as noise terms:


$$\begin{array}{c} I_{s}\left(0,\lambda \right)=I_{s}\left(0,\lambda_{B}\right)+I_{s}(0,\lambda \neq \lambda_{B})+I_{noise}=P_{s}\left(L,\lambda_{B}\right)exp\left(g_{B}P_{p}L_{eff}\right)+P_{s}(L,\lambda \neq \lambda_{B})+I_{noise} \end{array}$$ (14)

Here, $I_{s}\left(0,\lambda_{B}\right)$ and $I_{s}(L,\lambda \neq \lambda_{B})$ represent the in-band and out-of-band spectral components of the SUT, respectively. $I_{noise}$ denotes the noise terms generated by nonlinear effects within the fiber.

A fundamental limitation in Brillouin-based spectral analysis arises from the coexistence of amplified Stokes signals with unamplified out-of-band components propagating through the fiber. This spectral crosstalk degrades the measurement’s dynamic range. Separating weak spectral components from a strong baseline within the detected time-relative power signals poses a significant challenge. To address this, we employ a baseline correction algorithm to fit the unamplified signals and noise present in the raw measurement data. In practice, the algorithm first analyzes the characteristics of the unamplified components and noise in the data. By employing appropriate mathematical models, such as polynomial fitting, spline interpolation, or statistical methods, it estimates the signal baseline. The corrected signal is then obtained by subtracting the fitted baseline ${\tilde{I}}_{s}\left(0,\lambda \right)$ from the raw measurement data.


$$\begin{array}{c} {\tilde{I}}_{s}\left(0,\lambda \right)=I_{s}(L,\lambda \neq \lambda_{B})+I_{noise} \end{array}$$ (15)

After the unamplified signal and noise are fitted using baseline correction algorithms, the difference between the measured data and the baseline data, expressed as:


$$\begin{array}{c} I_{s}\left(0,\lambda \right)-{\tilde{I}}_{s}\left(0,\lambda \right)=P_{s}\left(L,\lambda_{B}\right)exp\left(g_{B}P_{p}L_{eff}\right) \end{array}$$ (16)

is proportional to the in-band components of the SUT at λ_B_. By sweeping the pump wavelength and measuring the backscattering signal, which varies according to the SUT’s spectral components across the wavelength range, the spectral profile of the SUT can be reconstructed.

The resolution of this method is intrinsically limited by the natural bandwidth of SBS. As is well known, within the low-gain region, the SBS gain spectrum exhibits a Lorentzian profile [1].


$$\begin{array}{c} g_{B}\left(\delta \omega^{\prime }\right)=\eta g_{\text{0}}\frac{(\Delta \omega_{B}/2{)}^{\text{2}}}{(\delta \omega {)}^{\text{2}}+(\Delta \omega_{B}/2{)}^{\text{2}}} \end{array}$$ (17)

Here, $\delta \omega^{\prime }=\omega -\omega_{p}-\omega_{D}$, $\omega_{p}$ denote the pump frequency, $\omega_{D}$ denote the Doppler frequency shift, $\Delta \omega_{B}$ and $g_{\text{0}}$ represent the full width at half height (FWHM) and peak gain of the Brillouin gain spectrum, respectively, η is the polarization impact factor (0 ≤ η ≤ 1). To overcome the limitation imposed by natural SBS bandwidth, we implement a two-stage SBS gain bandwidth compression technique [28]. The resulting composite gain coefficient is expressed as follows:


$$\begin{array}{c} G\left(\omega \right)=e^{2g_{\text{0}}-2A}=exp[\frac{2\eta g_{\text{0}}(\Delta \omega_{B}/2)}{(\omega -\omega_{\text{0}})+(\Delta \omega_{B}/2{)}^{\text{2}}}-2A] \end{array}$$ (18)

Here, A represents the inter-stage attenuation, and $\omega_{\text{0}}$ represent the frequency corresponding to the maximum Brillouin gain coefficient. Analogous to Eq (18), the resultant FWHM is given by:


$$\begin{array}{c} \Delta \omega_{BE}=\Delta \omega_{B}\sqrt{\frac{ln2}{2g_{\text{0}}-ln2}} \end{array}$$ (19)

**Fig 4:**
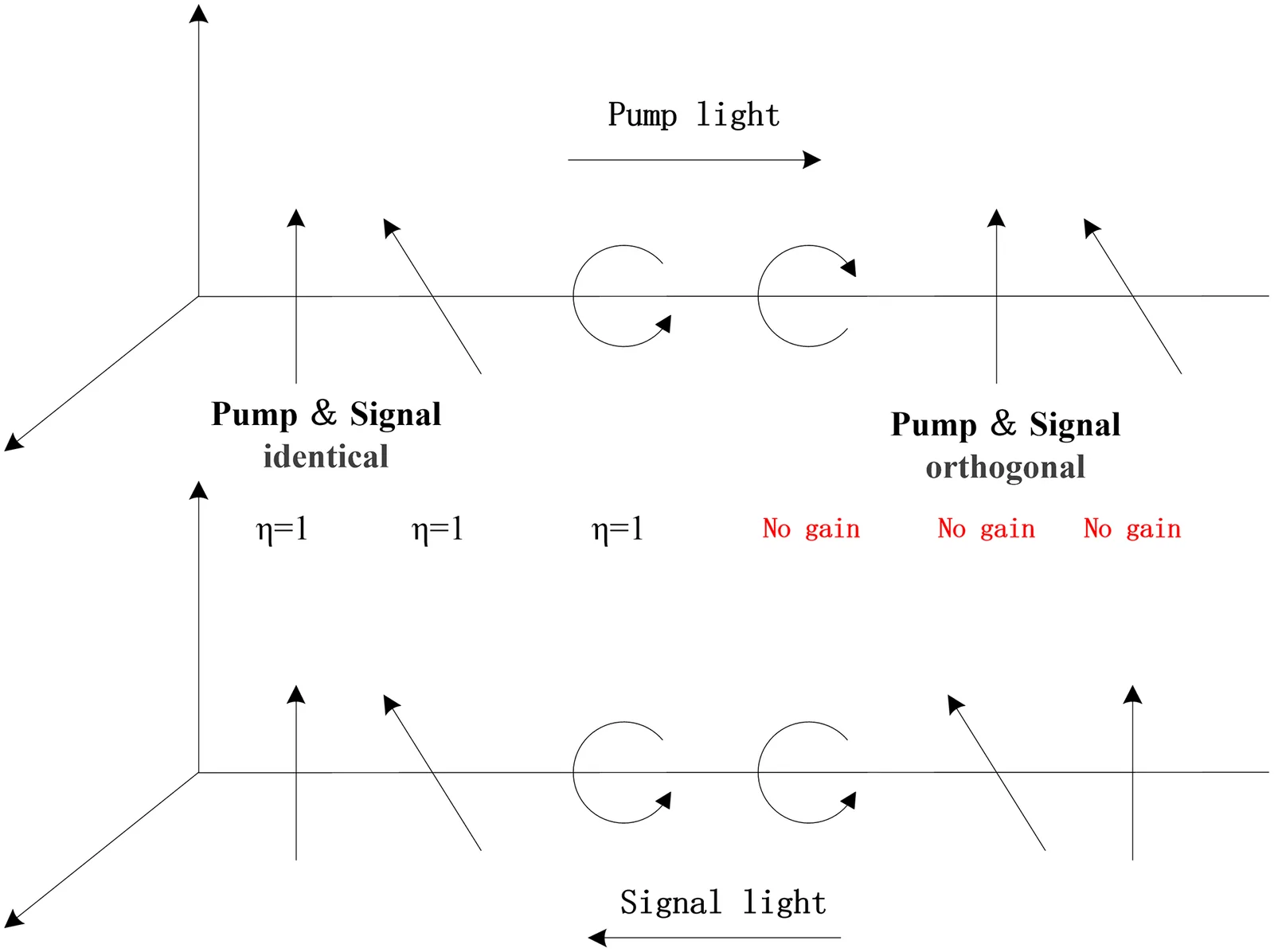
Polarization dependence of brillouin gain in optical fibers.


While bandwidth compression enhances spectral resolution, polarization sensitivity remains a significant challenge in Brillouin-based spectral analysis. As demonstrated in prior studies [29,30], the polarization-dependent gain is governed by the polarization impact factor η (0 ≤ *η* ≤ 1), which quantifies the degree of alignment between the relative SOPs of the pump and the SUT, as shown in Fig 4. Optimal gain (η = 1) occurs when the pump and signal are co-polarized, while orthogonal SOPs result in interaction (η = 0). When their SOPs are identical [29]:

$$\begin{array}{c} \eta_{∥}=\frac{\text{1}}{\text{2}}(1+s_{1p}s_{1s}+s_{2p}s_{2s}-s_{3p}s_{3s}) \end{array}$$ (20)

Here, ${\overset{⌢}{S}}_{s}=\left(s_{1s},s_{2s},s_{3s}\right)$ and ${\overset{⌢}{S}}_{p}=\left(s_{1p},s_{2p},s_{3p}\right)$ represent the normalized Jones vectors that characterize the polarization states of the SUT and pump signals, respectively, at a given location in the fiber.

If we now consider another pump wave with an orthogonal SOP, the polarization impact factor is expressed as:


$$\begin{array}{c} \eta_{⟂}=\frac{\text{1}}{\text{2}}(1-s_{1p}s_{1s}-s_{2p}s_{2s}+s_{3p}s_{3s}) \end{array}$$ (21)

According to the theoretical model established in the preceding chapters, adopting a dual orthogonal pump configuration by incorporating η_||_ and η_┴_, defined in Eqs. (20) and (21), eliminates the polarization-dependence of SBS-based filtering. The dual-pump scheme ensures that the Brillouin gain stabilizes at its theoretical maximum (η = 1) across temporal and spatial domains under all operating conditions.


$$\begin{array}{c} \eta_{∥}+\eta_{⟂}=1 \end{array}$$ (22)

The orthogonality between pump components is rigorously preserved in conventional single-mode fibers, as polarization transformations inherently maintain their unitary characteristics [31]. This fundamental property ensures compensation fidelity across extended propagation distances, guaranteeing the complementary nature of Brillouin amplification throughout the fiber’s entire span. By strategically replacing conventional single-pump configurations with polarization-multiplexed, wavelength-swept sources in the SBS process, we ensure that SBS gain instability, primarily caused by polarization fluctuations, is automatically compensated and eliminated, thereby enabling precise amplification of arbitrarily polarized SUTs.

This polarization-insensitive SBS filtering architecture exhibits significant potential for photonic and microwave-photonic applications, particularly in ultrahigh-resolution spectral analysis and optical signal processing. Its key merits are reflected in three distinguishing features: exceptional spectral resolution approaching fundamental physical limits, complete suppression of polarization-induced distortions, and improved long-term stability.

## Experiment and analysis

### Depolarizing setup by dual orthogonal pump

**Fig 5:**
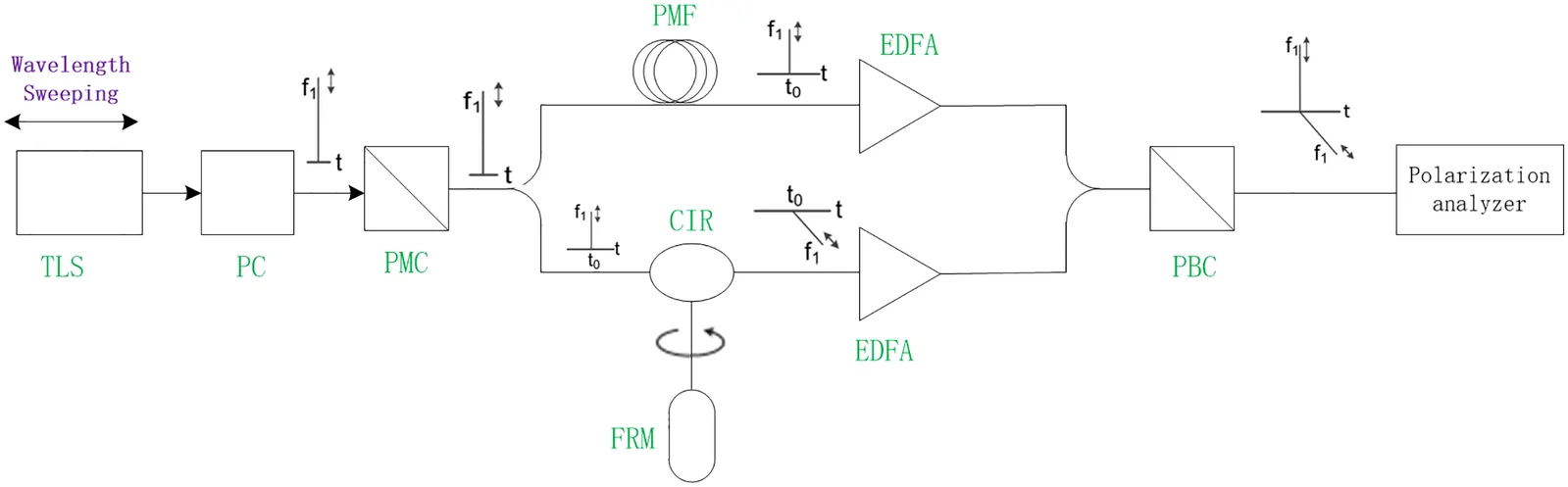
Schematic diagram of dual orthogonal pumps generated by the experimental setup. TLS, tunable laser source. PC, polarization controller. PMC, polarization-maintaining coupler. PMF, polarization-maintaining fiber. PMCIR, polarization-maintaining circulator. FRM, faraday rotator mirror. EDFA, erbium-doped fiber amplifier. PBC, polarization beam coupler.

Experimental validation of our theoretical model was conducted through rigorous characterization of the dual orthogonal pump architecture, with the detailed configuration schematically illustrated in Fig 5. Depolarization performance analysis revealed that the FRM integrated into a power-splitting MZI enables deterministic generation of orthogonally polarized pump components, a critical innovation to ensure the generation and preservation of polarization state orthogonality.

The optical experiments were conducted in a vibration-isolated optical laboratory maintained at ambient temperature of 25 ± 2°C and relative humidity of 50 ± 10% RH. Continuous-wave, linearly polarized light with wavelength (1525nm ~ 1560nm) and power of 5dBm from a TLS (Newport 8800) was split into two branches by a polarization-maintaining coupler (PMC). By employing a single TLS, we achieve stable measurements and precise pump control. The light in the lower branch passes through an FRM via a polarization-maintaining circulator, resulting in orthogonal polarization states between the input and output light. In contrast, the light in the upper branch traverses a 50 m polarization-maintaining fiber (YOFC Panda-type), ensuring its polarization state remains unaltered. Light from both branches is amplified by erbium-doped fiber amplifiers (EDFAs, Qnoptics EDFA-C + L) to 13dBm to achieve consistent and suitable optical power levels. The two branches, featuring orthogonal SOPs, are subsequently combined using a polarization beam coupler (PBC), forming dual orthogonal pumping light relative to the soon-to-be-defined pump wave at wavelength λ. The polarization controller (PC, General Photonics PLC-002) is not part of the dual orthogonal pump configuration; it is used to alter the SOP of the TLS output light for evaluating the depolarizer’s performance characteristics. A power meter (Keysight 81630B) and an optical spectrum analyzer (Yokogawa AQ6370E) were used for setup adjustments during testing.

By carefully matching the length of polarization-maintaining fiber (PMF) in the upper branch, time delays between the two paths are eliminated, ensuring no frequency offset exists between the two orthogonal light waves after they are combined. Consequently, the pump used for the SBS-based filter becomes wavelength-independent. The PMC also serves as a polarizer, ensuring the input light remains in a stable polarization state during the wavelength sweep executed by TLS. Thus, the pump for the SBS-based filter is free of polarization-sensitive components.

**Fig 6:**
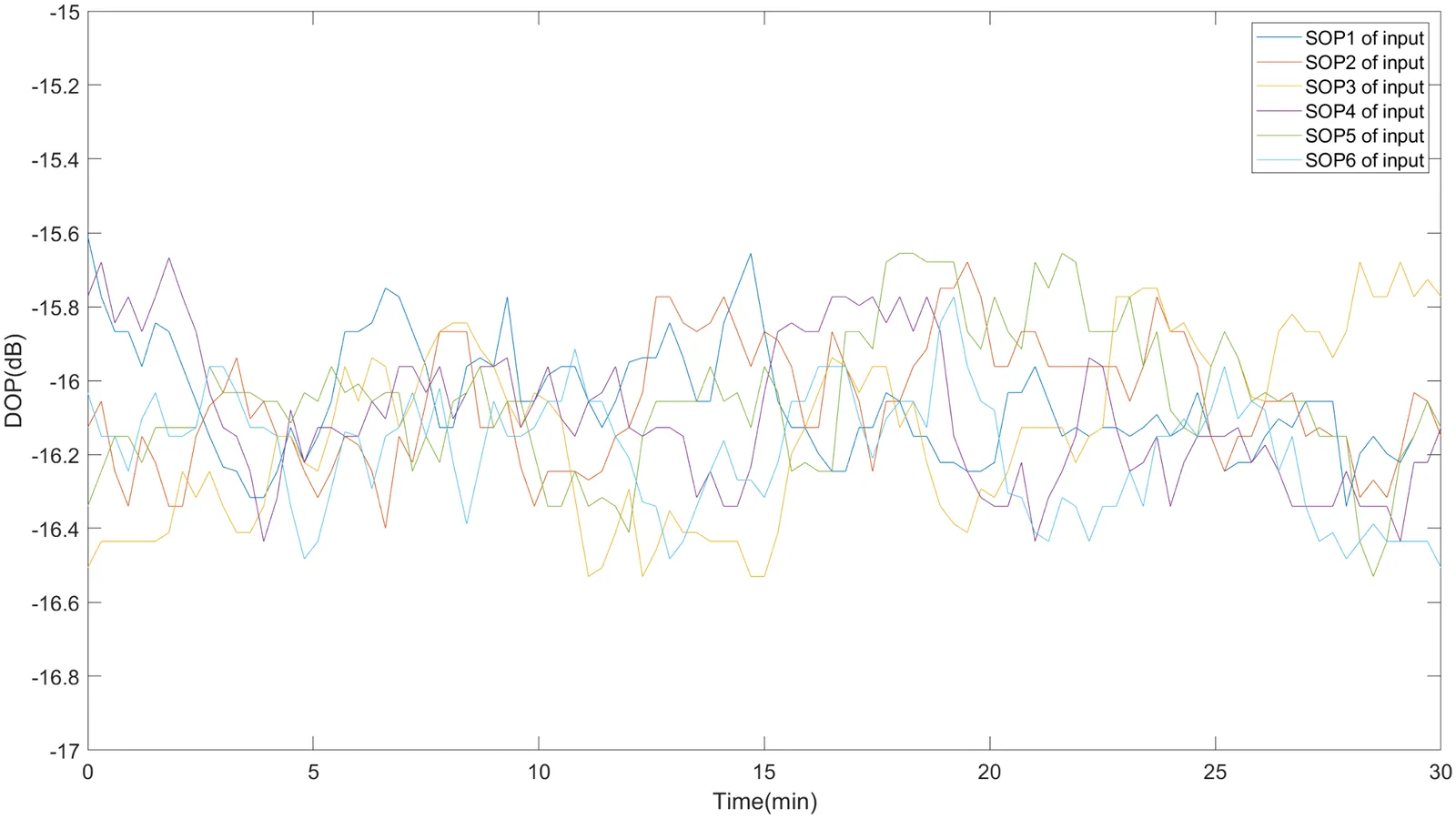
Measured DOP at the PBC output with different polarization state of the PMC input.

To quantify the depolarization efficacy, the DOP at the PBC output was measured using a polarization analyzer (General Photonics POD-101D). By systematically adjusting the SOP of the TLS output light with a polarization controller, the DOP measurements fluctuated within the range of −15.66 dB (center offset rate is 2.1%) to −16.45 dB (center offset rate is −2.8%), centered around −16 dB (equivalent to <±3%), over a period of 30 minutes across six distinct input polarization states, as shown in Fig 6. Notably, the depolarizer maintained this performance regardless of input SOP variations, confirming its polarization-independent operation and eliminating the need for active polarization control.

While this architecture simplifies system complexity by eliminating the need for polarization controllers, a particularly advantageous in multi-stage filtering schemes. It introduces a trade-off: the dual orthogonal pump configuration requires 1.5 times higher pump power compared to single-polarization schemes to achieve equivalent Brillouin gain. This redundancy stems from the dual-pump design, in which half of the optical power is distributed in each orthogonal component.

Despite this trade-off, the approach offers unique advantages over previous methods. First, regarding bandwidth compression, the two-stage SBS architecture effectively reduces the natural Brillouin bandwidth from approximately 20 MHz to around 10 MHz, enabling sharper filtering with a high degree of precision. By narrowing the Brillouin bandwidth, this approach can resolve fine spectral features more clearly providing more detailed information. Second, it ensures stable operation: the design achieves polarization-insensitive narrowband filtering while maintaining stable pump control and consistent filtering signal power. In many optical systems, polarization-related issues can significantly degrade signal quality. However, this method overcomes such problems, guaranteeing consistent performance regardless of the input signal’s SOP. Third, the approach offers dynamic tenability. The filter’s central frequency can be precisely adjusted by varying the pump wavelength, which is particularly useful for compensating for environmental perturbations such as temperature- or stress-induced Brillouin shifts. By adjusting the pump wavelength to counteract these shifts, the filter maintains optimal operation and ensures accurate signal processing. Fourth, it offers multi-dimensional flexibility: the proposed filter supports high-precision tuning across multiple parameters without compromising stability. Users can fine-tune various aspects of the filter, such as the bandwidth, center frequency, and gain, while maintaining a stable output. This multi-dimensional tunability provides greater control over the filtering process, making it adaptable to diverse application requirements.

### SBS-based ultrahigh-resolution OSA with pump depolarizing

Building on this depolarization method, we implemented a polarization-insensitive optical spectrum analyzer (OSA) that uses a wavelength-swept dual-pump architecture. This architecture enables the OSA to perform ultrahigh-resolution and stable measurements.

Conventional SBS-based filters are hindered by polarization-dependent gain variations, as reported in reference [1,32]. In contrast, our dual orthogonal-pump configuration effectively overcomes this limitation. By employing two orthogonal pump waves, the system is rendered insensitive to the SOP of the input signal, ensuring consistent gain regardless of polarization orientation. Furthermore, the wavelength-sweeping mechanism incorporated into our design suppresses four-wave mixing artifacts, typically associated with multi-tone schemes.

**Fig 7:**
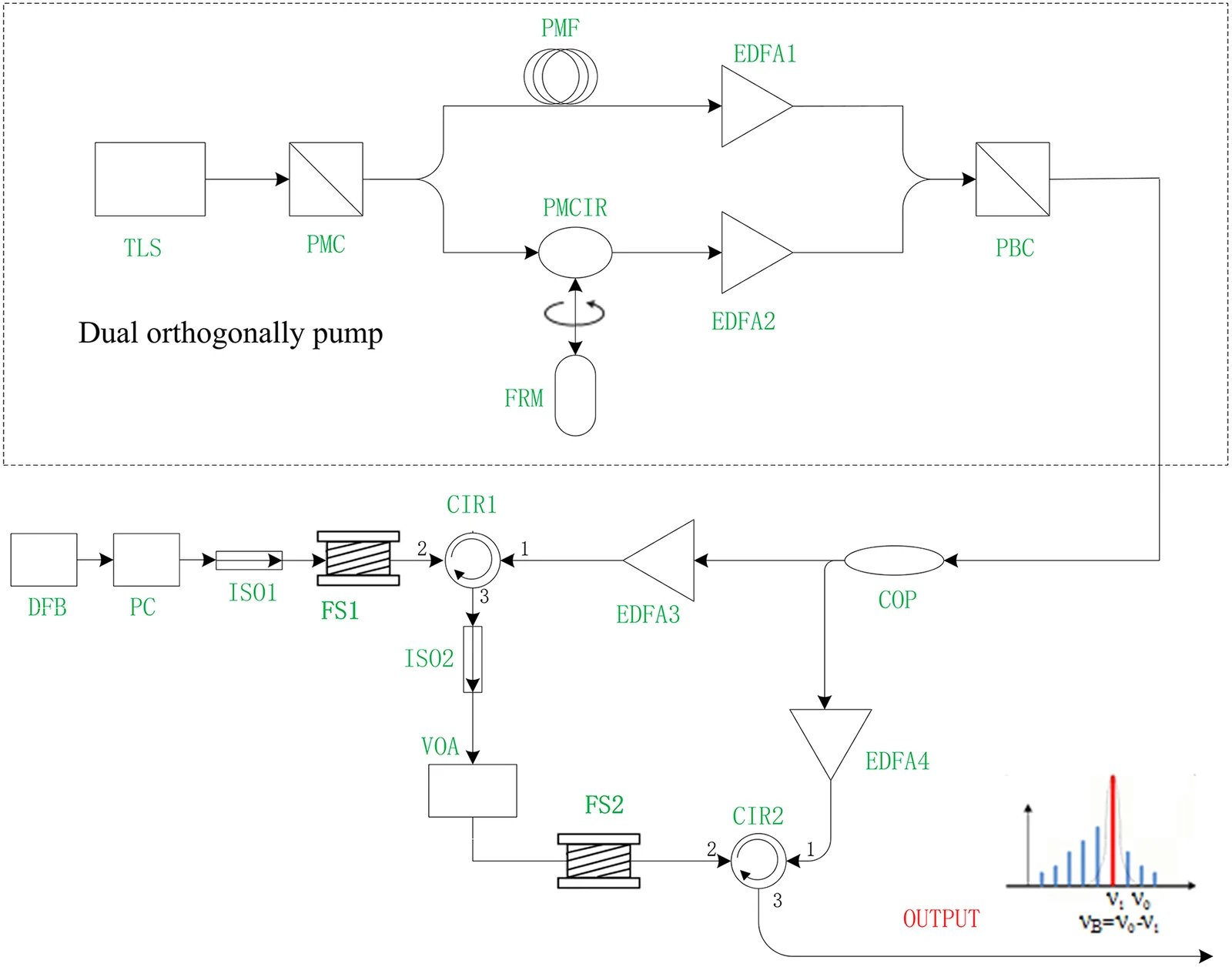
Schematic of SBS-based Ultra-high resolution OSA. TLS, tunable laser source. PC, polarization controller. PMC, polarization-maintaining coupler. PMF, polarization-maintaining fiber. PMCIR, polarization-maintaining circulator. FRM, Faraday rotator mirror. EDFA, erbium-doped fiber amplifier. PBC, polarization beam coupler. DFB, distributed feedback laser. ISO, isolator. FS, fiber spool. VOA, variable optical attenuator. CIR, circulator. COP, coupler.

The optical experiments were conducted in a vibration-isolated optical laboratory with an ambient temperature of 25 ± 2 °C and relative humidity of 50 ± 10% RH. Fig 7 illustrates the experimental setup, which incorporates standard telecommunications-grade optical components. At the core of this design is the depolarized pump, generated through the dual orthogonal configuration detailed previously. This pump is split into two beams by a coupler (COP) and then amplified within a two-stage SBS progression chain. To optimize system performance, each optical beam in the two-stage chain is amplified by an EDFA to 14dBm, with the EDFA operating under automatic power control. As multi-stage amplification improves pump efficiency and enhances spectral resolution [28], the two-stage design enables narrowband optical filtering by increasing pump power while maintaining filter linearity, thereby optimizing power efficiency with precision. The power meter, wavelength meter (Yokogawa AQ6151B) and optical spectrum analyzer were used for experimental setup adjustments during testing.

To maintain optimal operation, a variable optical attenuator (VOA) dynamically adjusts the first-stage output power, confining the SBS process within its linear gain regime [31,32]. This approach safeguards the fidelity of spectral filtering while accommodating power fluctuations. In the two-stage chain, the gain medium, a 1-km highly nonlinear fiber spool with an engineered Brillouin coefficient, has been optimized to support only fundamental optical and acoustic modes. This ensures selectivity in the single-peak Brillouin gain spectrum.

To conclusively validate the polarization-independence operation of the OSA, a PC was systematically employed to vary the SOP of the output from a distributed feedback laser (DFB) (NKT E15), which served as the SUT at 1dBm and 1549.8316nm. The output power of DFB was measured by a power meter and the output wavelength was measured by a wavelength meter. By carefully adjusting the PC in 0.2π radian increments, we conducted a quantitative characterization of OSA measurement fidelity under 10 distinct polarization conditions. This method enabled rigorous validation of the OSA’s polarization-insensitive operation across a wide range of polarization conditions.

**Fig 8:**
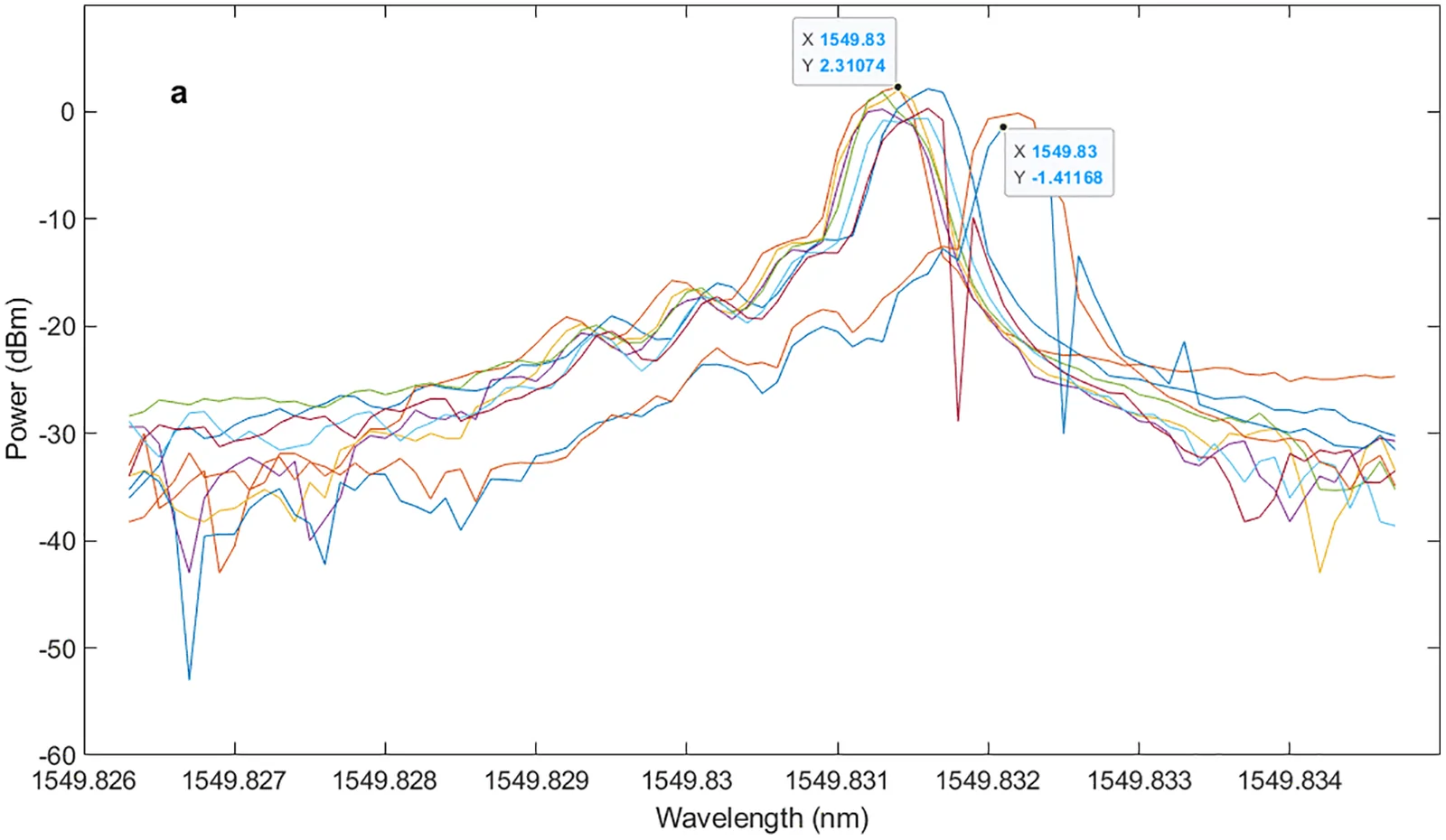
Measurement results with linear pumping source.

**Fig 9:**
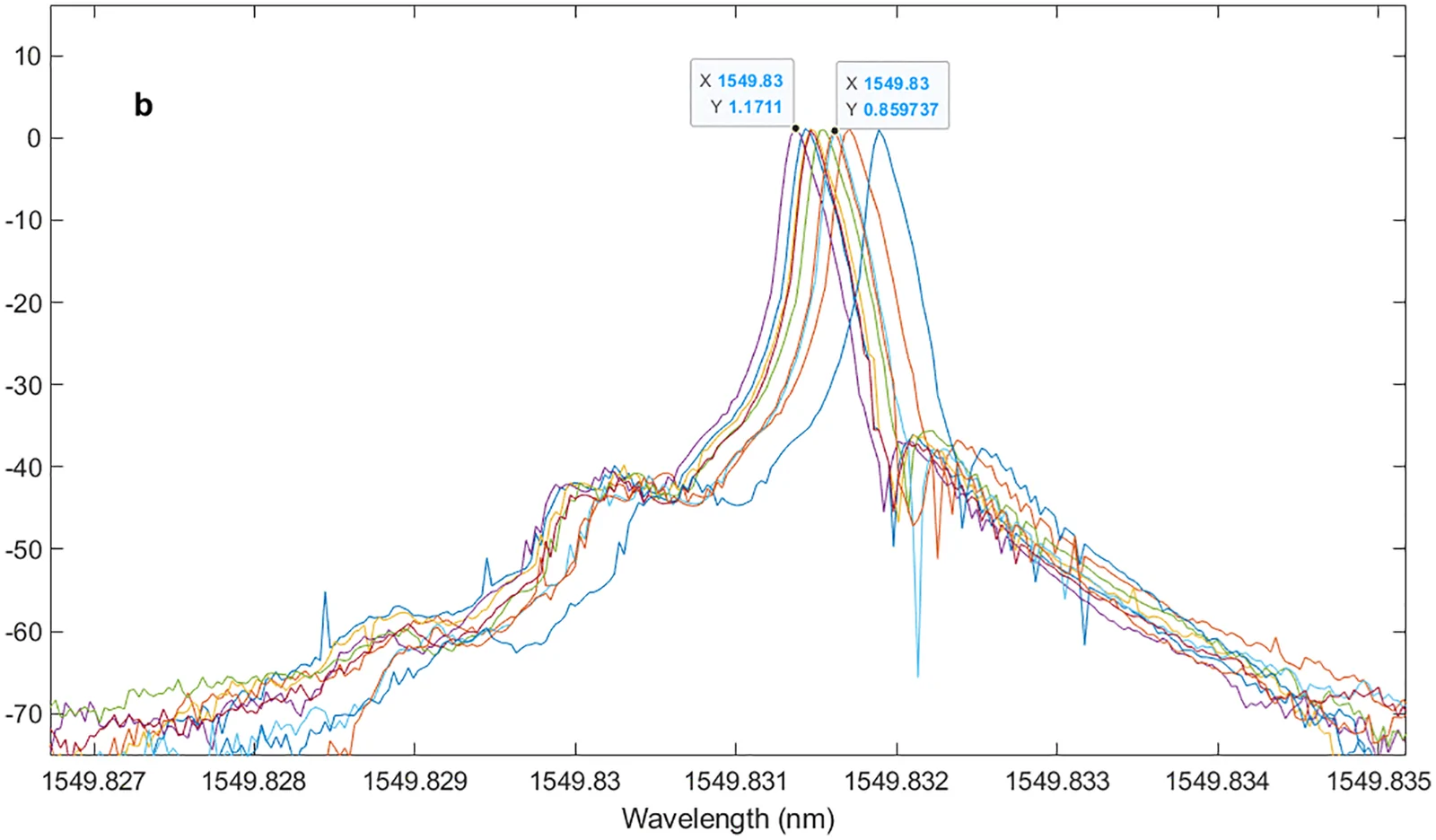
Measurement results with depolarizing pumping source.

To systematically validate the polarization-independence operation of the proposed SBS-based OSA, the SUT’s SOP was altered across a broad range of polarization conditions. We conducted a comparative characterization of measurement fidelity under two distinct pumping configurations: conventional single-polarization and our depolarized dual-pump architecture. As shown in Fig 8, the use of a conventional single-polarization pump source resulted in pronounced spectral distortions and significant fluctuations in both spectral shape and power. The peak optical power of the spectral curve fluctuated from −1.412 dBm to 2.311 dBm over ten repeated measurements, with an average peak optical power of 0.1911 dBm. The peak wavelength of the spectral curve varied from 1549.8313 nm to 1549.8323 nm over ten repeated measurements, with an average peak wavelength of 1549.83168 nm, a wavelength accuracy of ±0.7 pm, and a spectral power stability of ±2.4 dB. Comparing the spectral measurement results of the conventional single-polarization architecture corresponding to Fig 8 with those of the depolarized dual-pump architecture corresponding to Fig 9, the difference in the dynamic range of the measured spectral curves between these two cases exceeds 20 dB. Furthermore, considering that the pronounced polarization effect shown in Fig 8 leads to larger fluctuations in the spectral baseline, the power level of the spectral curve exhibited a polarization-dependent gain exceeding 20 dB, which severely impaired measurement accuracy by distorting the power spectral density. This instability underscores a fundamental limitation of conventional SBS-based systems, where misalignment between the pump and signal SOPs disrupts gain uniformity.

In stark contrast, Fig 9 demonstrates the performance of the proposed depolarized dual-pump architecture. Notably, the system maintained consistent spectral fidelity across all tested SOPs, reducing the polarization-dependent gain to less than 1 dB. The peak optical power of the spectral curve fluctuated from 0.8597 dBm to 1.171 dBm over ten repeated measurements, with an average peak optical power of 0.9936 dBm. The peak wavelength of the spectral curve varied from 1549.8313 nm to 1549.8318 nm over ten repeated measurements, with an average peak wavelength of 1549.83159 nm. This breakthrough enables ultrahigh-resolution measurements with unprecedented precision, achieving a wavelength accuracy of ±0.3 pm and a spectral power stability of ±0.2 dB. By eliminating polarization induced artifacts, the design enables reliable spectral analysis for arbitrarily polarized signals, overcoming a critical hurdle for practical applications in optical sensing and communications.

**Fig 10:**
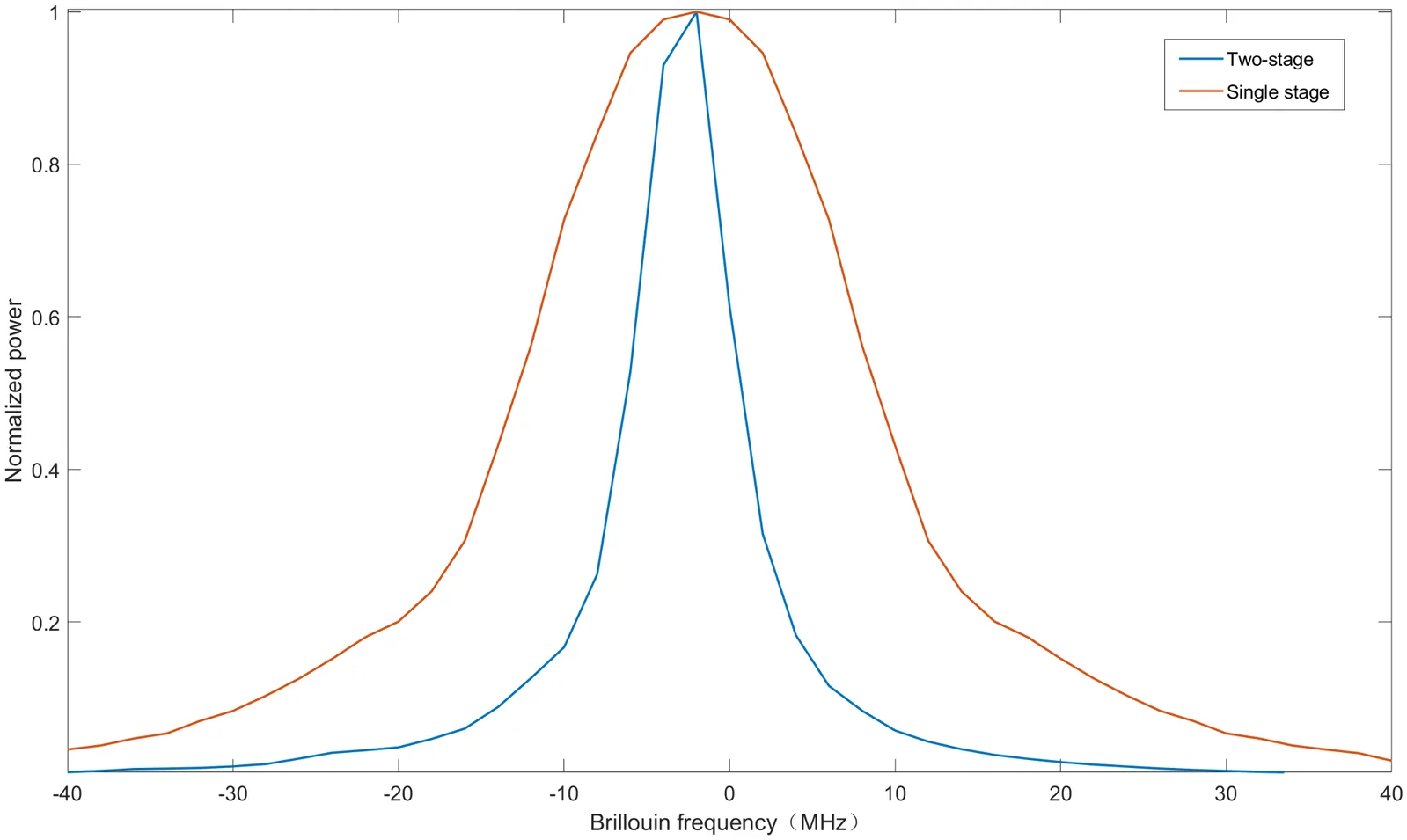
Measured normalized gain of SBS with single and two stage amplifying scheme.

To further quantify the performance of the proposed SBS-based OSA, we power-normalized and compared the measured spectra from single-stage and two-stage configurations, as depicted in Fig 10. The spectral shape and width of the SBS processes in the two-stage system are substantially improved. As illustrated by the red curve in Fig 10, the single-stage system exhibited a full width at half maximum (FWHM) of 17 MHz under high SOP alignment, which is an inherent limitation of conventional scalar SBS processes. In contrast, as shown by the blue curve in Fig 10, the two-stage architecture reduced the FWHM to 10 MHz, demonstrating a remarkable 41% bandwidth compression. This enhancement can be attributed to the combined effects of dual-stage amplification and polarization discrimination enabled by the dual orthogonal pump configuration. The dual orthogonal pump configuration sharpens spectral selectivity by effectively attenuating components near the SBS gain line. This not only improves the resolution of the OSA but also enhances its ability to distinguish between closely spaced spectral features, making it more suitable for applications requiring high-precision spectral analysis.

Crucially, this enhancement directly translates into superior dynamic range and noise performance. By suppressing off resonance spectral artifacts, the two-stage system attains a filter selectivity of 80 dB, a critical advancement for high-precision applications that enables clear discrimination of closely-spaced spectral lines. Notably, the optimized polarization discrimination also significantly minimizes noise contributions, ensuring robust performance in practical sensing and communication scenarios.

**Fig 11:**
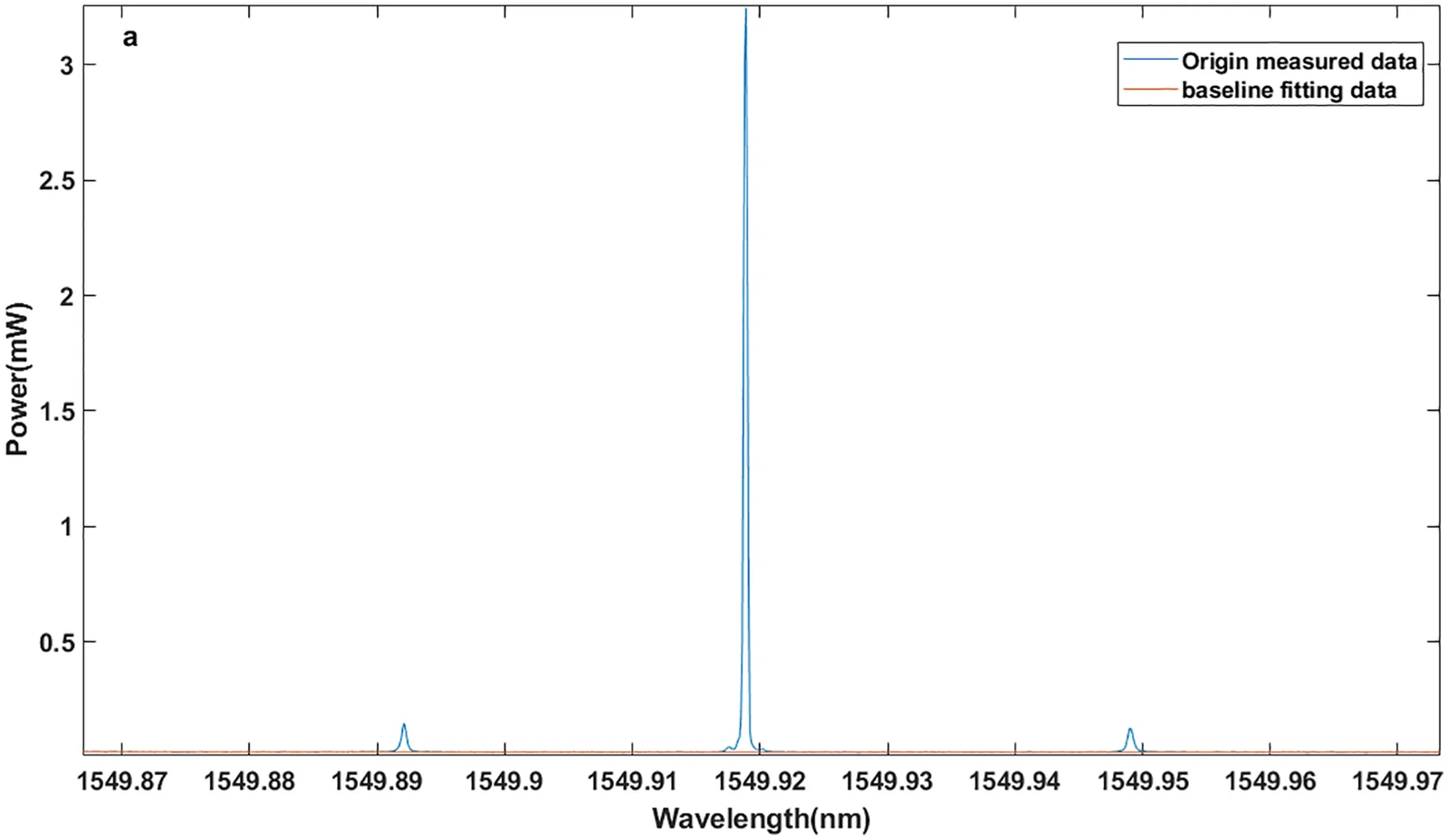
Measured data and baseline fitting result.

**Fig 12:**
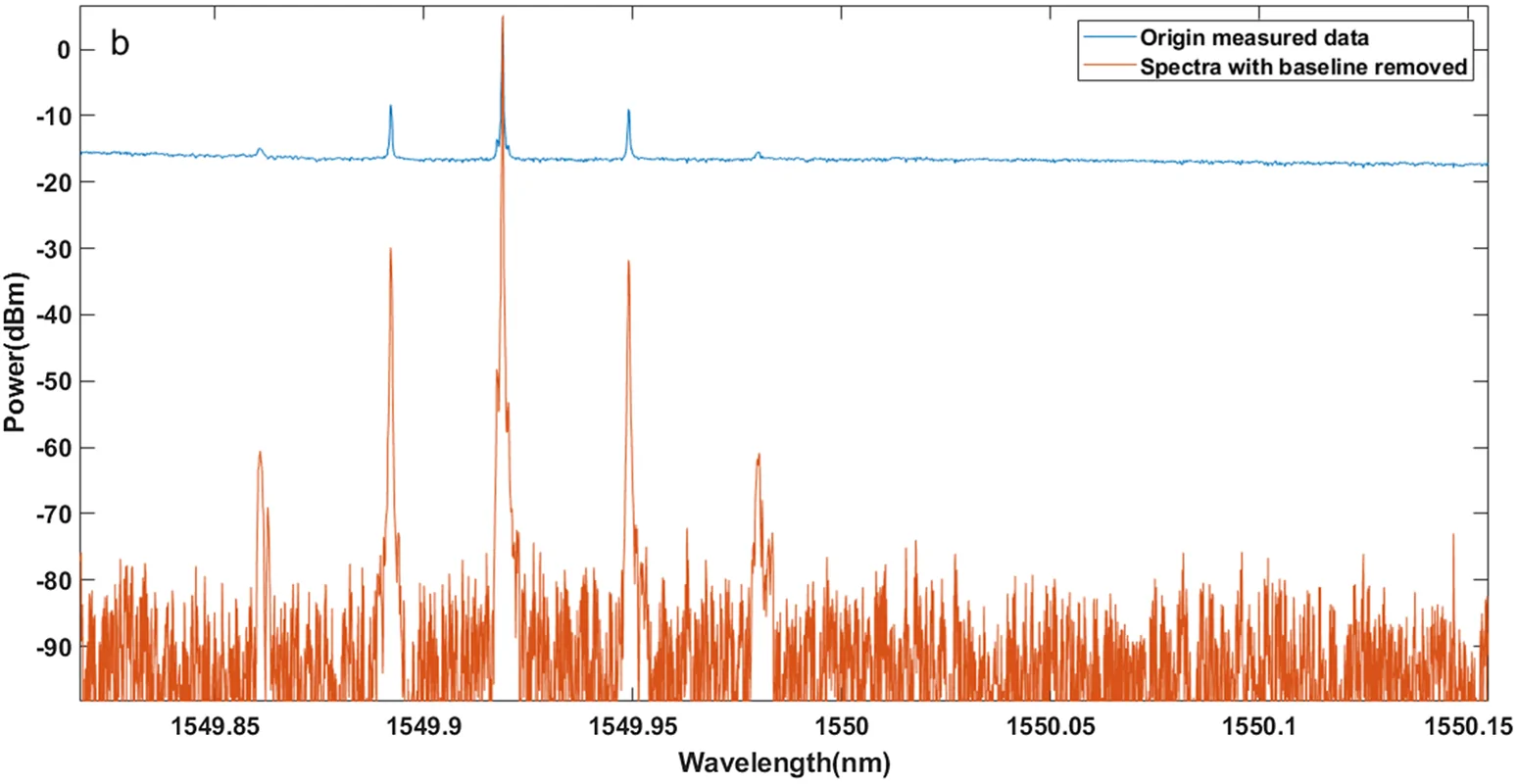
Precision spectra before and after baseline removal with dynamic range improvement.

A critical challenge in the experimental data stemmed from a dominant baseline signal. This baseline comprised unamplified out-of-band components, Rayleigh scattering, and amplified spontaneous emission (ASE) during SBS amplification, as established by the theoretical model in preceding chapters. This severely restricted the dynamic range. To overcome these limitations, we implemented an adaptive iteratively reweighted penalized least squares (airPLS) [33] baseline correction algorithm. As depicted in Fig 11 and Fig 12, measured spectral data from an FP laser (Keysight 81606A) containing four side-mode sidelobes is used to demonstrate the baseline correction process and its effect. The output power of FP laser is set to 5dBm.This algorithm functions by fitting the baseline to the spectral data and then subtracting it, thereby mitigating the impact of undesired components and compensating for nonlinear distortions. By applying the baseline correction algorithm, we can effectively remove this baseline, revealing the true spectral characteristics.

The origin measured raw data, represented by the blue curve in Fig 11 and Fig 12, exhibited a compressed dynamic range of approximately 19 dB, obscuring critical spectral details. The baseline-fitted data, represented by the red curve in Fig 11, revealed that baseline drift was the dominant factor contributing to this limitation. After baseline removal and power calibration, as shown by the red curve in Fig 12, the processed spectra achieved a true dynamic range of 80 dB, underscoring the algorithm’s efficacy. The baseline correction algorithm can achieve a dynamic range improvement of nearly 60 dB by removing the constant component of the measured spectral curve. Notably, the residual post-correction noise floors were determined by conventional photodetector and electronic noise mechanisms, confirming that baseline drift, rather than fundamental system noise, was the primary constraint.

This refinement clearly demonstrates that the baseline correction algorithm enables high-fidelity spectral recovery. Importantly, this approach unlocks the OSA’s full measurement potential while maintaining compatibility with standard optoelectronic hardware. This compatibility is crucial, as it allows for seamless integration into existing optical systems without necessitating major overhauls to the hardware infrastructure.

## Discussion

As demonstrated by the experiments, the SBS depolarization method proposed in this paper offers significant practical benefits for real-world systems. In spectral analysis applications, a wavelength accuracy of ±0.3 pm and a spectral power stability of ±0.2 dB enable the OSA to achieve performance comparable to that of a wavelength meter and an optical power meter. This capability allows for direct observation and analysis of fine structures in modulated optical signals within high-speed optical communication systems, dramatically accelerating both fundamental research and practical deployment of high-speed optical communication technologies. In distributed sensing, an 80 dB dynamic range allows for longer sensing distances (>80km) and higher spatial resolution (<1m). In on-chip nonlinear optics, a 10 MHz resolution enables the generation of multiline (>30) Brillouin frequency combs. In microwave-photonic application, an 80 dB dynamic range and 10 MHz resolution enable the division of wideband signals (>GHz) in different frequencies and bandwidths into parallel narrowband (<100MHz) signals for related processing with low-frequency devices.

## Conclusions

In summary, we demonstrate a polarization- and wavelength-independent SBS-based filter that leverages dual orthogonally polarized pumps to suppress birefringence-induced polarization fluctuations in optical fibers. Central to this achievement, we first developed a theoretical framework for modeling polarization-dependent SBS dynamics and subsequently proposed a novel dual-pump architecture. This approach enables complete mitigation of polarization-dependent gain variations during SBS amplification, resulting in a stable, narrowband tunable optical filter with a resolution of 10 MHz and polarization-insensitive characteristics. Notably, we validated its application in ultrahigh-resolution optical spectral analysis. For arbitrarily polarized input signals, we achieved spectral measurements with a wavelength precision of ±0.3 pm, power stability of ±0.2 dB, and sweep speeds of 20 nm/s. Future work will focus on the application and transformation of these experimental results. Specifically, based on the current experimental state of ultrahigh-resolution spectral measurements, we will proceed with the design and commercialization of a high-resolution optical spectrum analyzer (HOSA). Furthermore, we will develop a series of HOSAs tailored to different operating wavebands and application scenarios. We believe that such OSAs hold promising prospects in fields such as optical communications, optical materials and photonic devices, and quantum optics, among others.

Beyond its immediate performance metrics, this platform offers not only improved narrowband filtering, high-resolution spectral analysis, and stable gain characteristics but also paves the way for more robust and versatile SBS-based solutions. However, the dual orthogonal pump configuration demands higher pump power, and the limited tuning range of resolution may affect its applications in fields such as photonic integration. Therefore, future research efforts will focus on investigating novel optical filtering principles and developing practical filter architectures to achieve spectral filtering with both lower pump power requirements and reconfigurable bandwidths spanning from kHz to GHz.

These advancements unlock a wealth of new opportunities in optical communications (e.g., polarization-robust signal processing), optical storage (e.g., optical delay, slow and fast light generation), distributed fiber sensing (e.g., strain and temperature mapping in unshielded environments), microwave photonics (e.g., RF filtering and beamforming), on-chip integrated photonics (e.g., photonic control and generation), and optical neural networks (e.g., integrated communication, computing and sensing), among others.
